# Genome Sequence of *Rhodococcus erythropolis* Strain CCM2595, a Phenol Derivative-Degrading Bacterium

**DOI:** 10.1128/genomeA.00208-14

**Published:** 2014-03-20

**Authors:** Hynek Strnad, Miroslav Patek, Jan Fousek, Juraj Szokol, Pavel Ulbrich, Jan Nesvera, Vaclav Paces, Cestmir Vlcek

**Affiliations:** a Institute of Molecular Genetics, Academy of Sciences of the Czech Republic, Prague, Czech Republic; b Institute of Microbiology, Academy of Sciences of the Czech Republic, Prague, Czech Republic; c Department of Biochemistry and Microbiology, Institute of Chemical Technology, Prague, Czech Republic

## Abstract

We announce the completion of the genome sequence of a phenol derivative-degrading bacterium, *Rhodococcus erythropolis* strain CCM2595. This bacterium is interesting in the context of bioremediation for its capability to degrade phenol, catechol, resorcinol, hydroxybenzoate, hydroquinone, *p*-chlorophenol, *p*-nitrophenol, pyrimidines, and sterols.

## GENOME ANNOUNCEMENT

Members of the genus *Rhodococcus* possess a wide range of metabolic capabilities applicable for biodegradation of diverse environmental pollutants (1, 2) and for various biotransformations (3, 4). The strain *Rhodococcus erythropolis* CCM2595 (NCIB8147; JCM3132; ATCC 11048) was isolated from soil. Originally, it was classified as a strain of the species *Jensenia canicruria* (5). Later, it was reclassified into the species *Rhodococcus erythropolis* (6). *R. erythropolis* CCM2595 has been shown to utilize phenol, catechol, resorcinol, hydroxybenzoate, hydroquinone, *p*-chlorophenol, *p*-nitrophenol (7), pyrimidines (8), and sterols (9) as carbon sources. In addition to various metabolic activities, some of its characteristics, e.g., resistance to toxic compounds and biofilm formation, have proven useful in the biotechnological industry (10). A host-vector system has been developed for the strain (11), and the methods of genetic manipulation within its chromosome have been established (7). The development of genetic techniques have enabled detailed analysis of the *R. erythropolis* CCM2595 *catRABC* gene cluster, which codes for the enzymes of the catechol degradation pathway (12), and construction of recombinant plasmid-carrying *R. erythropolis* CCM2595 derivatives, which exhibit even more efficient phenol degradation in industrial wastewaters (12). *R. erythropolis* CCM2595 has also been used for directed biosynthesis of triacylglycerols containing branched-chain fatty acids (13) and ω-phenyl fatty acids (14).

The genome of *R. erythropolis* CCM2595 was sequenced using 454 GS-FLX technology (15). Whole-genome shotgun sequencing produced 244,559,207 bp of sequencing data in 582,471 reads. The reads were assembled using Newbler 2.5.3 (454 Life Sciences) into 44 contigs with an *N*_50_ length of 374,893 bp and an average coverage of 38.3×. All sequencing gaps were closed in Consed 19 (16). The complete genome consists of one circular chromosome (6,281,198 bp) and one circular plasmid (90,223 bp), which is already known as pRECF1 (17). Both replicons have a relatively high GC content of 62.5%.

The complete sequence was searched for putative protein-coding genes using Critica (18), Prodigal (19), and Glimmer (20). Aragorn (21) and tRNAscan (22) were used to localize tRNA and transfer-messenger RNA (tmRNA) genes, and RNAmmer (23) was employed to find rRNA and noncoding RNA (ncRNA) genes. The functions of the predicted protein-coding genes were assigned by the PGAAP pipeline (http://www.ncbi.nlm.nih.gov/genome/annotation_prok/). The annotation results were combined and verified within Artemis (24). In total, 5,830 predicted coding regions (CDSs), 12 rRNAs, 53 tRNAs, 1 tmRNA, and 5 ncRNAs were predicted and annotated.

Based on our results, we anticipate that *R. erythropolis* strain CCM2595 will display rich and complex metabolic capabilities, far beyond the utilization of benzene derivatives or catechol metabolism originally associated with this strain (7, 12).

### Nucleotide sequence accession numbers.

The genome sequences were deposited at DDBJ/EMBL/GenBank under the accession numbers CP003761 (chromosome) and CP003762 (plasmid pRECF1).
